# An Infrared Actin Probe for Deep-Cell Electroporation-Based Single-Molecule Speckle (eSiMS) Microscopy

**DOI:** 10.3390/s17071545

**Published:** 2017-07-01

**Authors:** Sawako Yamashiro, Naoki Watanabe

**Affiliations:** 1 Laboratory of Single-Molecule Cell Biology, Kyoto University Graduate School of Biostudies, Kyoto 606-8501, Japan; watanabe.naoki.4v@kyoto-u.ac.jp; 2 Department of Pharmacology, Kyoto University Graduate School of Medicine, Kyoto 606-8501, Japan

**Keywords:** single-molecule imaging, actin dynamics, near infrared probe

## Abstract

Single-molecule speckle (SiMS) microscopy is a powerful method to directly elucidate biochemical reactions in live cells. However, since the signal from an individual fluorophore is extremely faint, the observation area by epi-fluorescence microscopy is restricted to the thin cell periphery to reduce autofluorescence, or only molecules near the plasma membrane are visualized by total internal reflection fluorescence (TIRF) microscopy. Here, we introduce a new actin probe labeled with near infrared (NIR) emissive CF680R dye for easy-to-use, electroporation-based SiMS microscopy (eSiMS) for deep-cell observation. CF680R-labeled actin (CF680R-actin) incorporated into actin structures and showed excellent brightness and photostability suitable for single-molecule imaging. Importantly, the intensity of autofluorescence with respect to SiMS brightness was reduced to approximately 13% compared to DyLight 550-labeled actin (DL550-actin). CF680R-actin enabled the monitoring of actin SiMS in actomyosin bundles associated with adherens junctions (AJs) located at 3.5–4 µm above the basal surfaces of epithelial monolayers. These favorable properties of CF680R-actin extend the application of eSiMS to actin turnover and flow analyses in deep cellular structures.

## 1. Introduction

Single-molecule fluorescence microscopy is one of the most sensitive detection techniques, and enables the visualization of individual molecules in cells. Live-cell single-molecule imaging has provided critical information on remodeling of the actin cytoskeleton [1,2,3], the motion of plasma membrane proteins [4,5], dynamics of microtubule motor proteins [6,7], and so on. The most accessible observation region for single-molecule imaging is the cell periphery and the area near the plasma membrane where background fluorescence can be reduced. On the other hand, there is a growing trend of extending the imaging approach to three-dimensional cellular structures by the development of new illumination systems. For example, highly inclined thin illumination (HILO) illuminates a thin section that is several microns deep in the sample [8]. The HILO technique has been used to observe targeting of chromatin regulatory complexes to chromatin [9], dynamics of transcription factors in the nucleus [10], and single-molecule dynamics near the cell cortex of *Caenohabditis elegans* embryos [11]. Gabhardt et al. developed reflected light-sheet microscopy, which enables selective plane illumination of a thin light sheet of ~1 µm, and analyzed transcription factor–DNA interactions in the nucleus [12]. Lattice light-sheet microscopy achieves high image acquisition speed with frame rates more than 200 slices per second with a thin light-sheet illumination [13]. These selective plane imaging light-sheet microscopies for deep inside the cell are powerful technologies to monitor fast processes such as diffusion of molecules in the nucleus. On the other hand, various endogenous fluorophores including NADH, NADPH, flavins, lipofuscin-like lipopigments and vitamin A exist in the cytoplasm, which causes strong autofluorescence especially in the perinuclear region [14]. Furthermore, autofluorescence emission peaks of most endogenous fluorophores are 400–600 nm [14], which overlaps those of fluorophores employed for single-molecule imaging, such as EGFP [4,6,11,15], tetramethylrhodamine [13], DyLight 549/550 [16,17], Dendra2 [10] and mEos2 [12]. Those issues of autofluorescence have hampered single-molecule imaging in the view field near the area emitting strong autofluorescence in the cytosplasm.

We recently developed a new, electroporation-based single-molecule speckle (eSiMS) microscopy [16,17] (also see our accompanying review). In this method, we employed an actin probe that is chemically labeled with a fluorescent DyLight 549 dye on lysine side chains. Electroporation-mediated delivery of DyLight 549-actin (DL549-actin) into cells enables the labelling of cells with 100% efficiency at the optimal low density suitable for SiMS microscopy. Using near infrared (NIR) emissive organic dyes extends eSiMS microscopy with fluorescence imaging in the NIR region where the background generated by cellular autofluorescence is low. Furthermore, NIR dyes are suitable for imaging in thick cells and tissues because fluorescence imaging in the long-wavelength region facilitates low phototoxicity to cells and deeper tissue penetration [18,19]. In this study, we introduce a new NIR actin labeled with CF680R dye for eSiMS microscopy and its application to single-molecule imaging of actin in cell–cell adhesion with epi-fluorescent microscopy.

## 2. Materials and Methods

### 2.1. NIR-Labeled Actin

Rabbit skeletal muscle actin labeled with DyLight649-NHS ester (Thermo Fisher Scientific: Waltham, MA, USA), CF680R-NHS ester (Biotium: Fremont, CA, USA), CF750-NHS ester (Biotium) and CF770-NHS ester (Biotium) were prepared as described previously [16,17].

### 2.2. Plasmids

The expression vector harboring the defective CMV promoter (delCMV) for Lifeact-mCherry was described previously [20]. The expression vectors for human vinculin tagged with EGFP at the N-terminus were described previously [16]. Human MRLC cDNA [21] was subcloned into a pTagRFP-T-C.

### 2.3. Cell Culture and Electroporation

XTC cells were maintained as described previously [15,22]. A6 cells were maintained as described previously [23]. XTC cells and A6 cells were subjected to electroporation to deliver NIR-labeled actin as described previously [16,17].

### 2.4. Single-Molecule Speckle Imaging and Data Analysis

SiMS imaging and live-cell imaging were carried out in XTC cells as described previously [17,22]. Briefly, XTC cells were allowed to spread on a poly-L-lysine-coated coverslip in 70% L-15 Leibovitz medium (Invitrogen: Carlsbad, CA, USA) without serum. A6 cells were allowed to spread on a collagen (BD Biosciences: San Jose, CA, USA)-coated coverslip in 50% L-15 Leibovitz medium without serum. To observe CF680R-actin in epithelial A6 monolayers, A6 cells were seeded at a density of 1 × 10^5^ cells/cm^2^ on a collagen-coated coverslip and maintained in 50% L-15 Leibovitz medium with 10% fetal bovine serum for 48 h. Imaging was performed using a microscope (IX83, Olympus: Tokyo, Japan) equipped with 75 W xenon illumination and a cooled EMCCD camera (Evolve 512, Photometrics: Tucson, AZ, USA), or a microscope (IX71, Olympus) equipped with 100 W mercury illumination and a cooled EMCCD camera (Evolve 512; Photometrics). A PlanApo 1.40 NA 100× oil objective (Olympus) was used to observe spread cells on a coverslip, and a UPLSAPO60XS2 1.30 NA 60× silicone immersion objective (Olympus) was used to observe actin structures associated with adherens junctions (AJs). Speckles were analyzed by using Speckle TrackerJ as described [16,17].

## 3. Results

**Figure 1:**
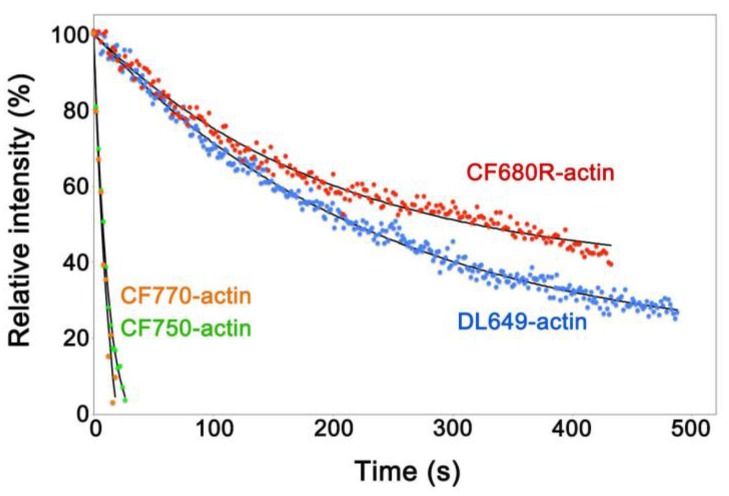
Intracellular photostability of fluorescent dye-labeled actin probes. XTC cells loaded with each fluorescent dye-labeled actin by electroporation were continuously exposed to excitation light using the 100 W mercury illumination system. The single-molecule speckle (SiMS) of each actin probe was acquired with 2 s exposure time without interval. The excitation light used was full for CF750- (green dots) and CF770-actin (orange dots), attenuated to 25% for DL649-actin (blue dots) or 12% for CF680R-actin (red dots). The filter sets used were the Cy5 filter set (Semrock: Rochester, NY, USA) for DL649-actin, the Cy5.5-C filter set (Semrock) for CF680R-actin and the IRDYE 800 filter set (Semrock) for CF750- and CF770-actin. Black lines show the single-exponential curve fitted to the data. Relative intensity (*y-*axis) shows percentages derived from the fluorescence intensity of the peripheral region of the XTC cell divided by the value at the *y-*intercept of the fitted curve. The data shown are representative of at least two independent experiments.

To develop a NIR actin probe for eSiMS, we prepared actin labeled at lysines with DyLight 649, CF680R, CF750 and CF770 dyes, respectively. These actin probes were delivered into the cytoplasm of *Xenopus* XTC cells by the electroporation method [16,17]. We compared photostability of the actin probes in the cell peripheral region to exclude autofluorescence in the cell body. In XTC cells, CF680R-labeled actin (CF680R-actin) and DyLight 649-labeled actin (DL649-actin) were considerably photostable, whereas actin labeled with CF750 or CF770 dyes was photobleached immediately after the first image (Figure 1, Video S1). Brightness of CF680R-actin was similar to that of DL649-actin. In the following study, we employed CF680R-actin as a NIR actin probe for eSiMS, because CF680R-actin is excitable with longer wavelength than DL649-actin.

**Figure 2:**
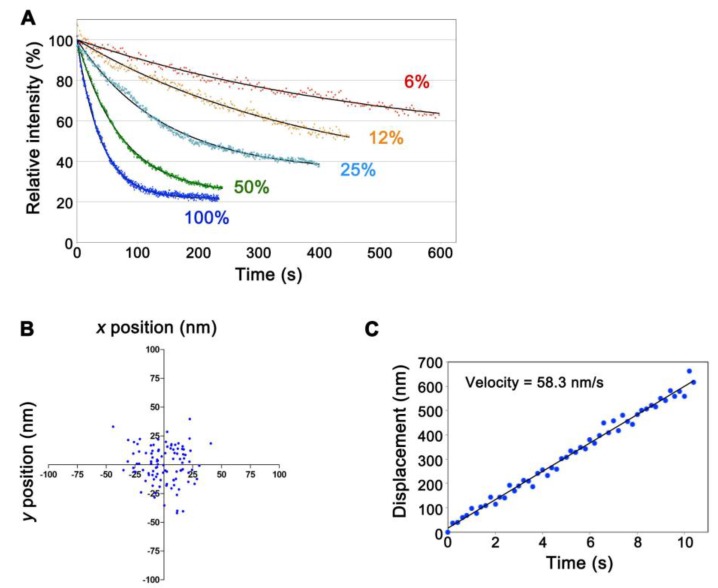
Characterization of CF680R-actin in live XTC cells. (**A**) Photostability of CF680R-actin using excitation light with the 75 W xenon illumination system in live cells. The excitation light used was full (100%) or attenuated to 50%, 25%, 12% or 6% as indicated. Black lines show the single-exponential curve fitted to the data. The data shown are representative of three independent experiments; (**B**) Dispersion of the central position of the immobile glass-bound CF680R-actin speckle acquired with a 200 ms exposure time and a full 75 W xenon excitation (fast-tracking images, 100 frames). The standard deviations of the centroid positions in one direction are 16.3 nm for the *x*-axis and 16.0 nm for the *y*-axis. The data shown are representative of the data from three independent experiments; (**C**) Displacement plot of the central position of CF680R-actin in lamellipodia in the series of fast-tracking images (50 frames). Note that CF680R-actin speckles moved with the retrograde actin flow in lamellipodia. The velocity of the CF680R-actin speckles was calculated from a linear fit. The data shown are representative of the data from three independent experiments.

We replaced the excitation filter (FF02-655/40-25, transmission wavelength 635–675 nm, Semrock) of the Cy5.5-C filter set (Semrock) for that with narrower transmission wavelength 651–671 nm (FF01-661/20-25, Semrock) to minimize autofluorescence. Both the 75 W xenon and the 100 W mercury illumination systems worked for SiMS imaging of CF680R-actin. The xenon illumination was 4- to 5-fold stronger in visualizing single-molecule CF680R-actin than the mercury illumination. To achieve visualization of CF680R-actin SiMS with a 1 s exposure time, the xenon excitation was attenuated to 6–12% using neutral density filters whereas the mercury excitation was attenuated to 25–50%. Photobleaching of CF680R-actin was slow under these conditions. Continuous exposure of the whole cell area to 6% xenon excitation for 200 s, 400 s and 600 s reduced CF680R-actin fluorescence to 82.0%, 73.1% and 62.9%, respectively (Figure 2A, Video S2). Therefore, CF680R-actin can be used to track actin SiMS for several minutes without a large influence of photobleaching. The brightness and photostability of CF680R-actin was comparable to those of DL549- or DL550-actin. The mercury illumination, which has a strong peak at the wavelength of around 546 nm, is suitable for SiMS imaging of DL549-actin and can be attenuated to 1.5% to visualize DL549-actin speckles [16]. Continuous exposure of the whole cell area to 1.5% of the mercury excitation for 600 s reduces DL549-actin fluorescence to only 76% of the original value [16].

We next examined whether CF680R-actin enables nanometer-scale displacement measurement as we reported with DL549-actin previously [16]. In the nanometer-scale displacement analysis, DL549-actin achieves low localization error of 8–8.5 nm and measurement of the speed of the actin flow within 100–150 nm that is below the diffraction limit of the light microscope in live cells [16]. The unattenuated 75 W xenon illumination was used to monitor CF680R-actin speckles with a high signal-to-noise ratio. We acquired images at 200 ms intervals (fast tracking) for the short duration of 10 s. Subpixel localization of the centroids of CF680R-actin SiMS was determined by using the two-dimensional Gaussian fit model of Speckle TrackerJ [16]. Under this condition, the centroids of immobile CF680R-actin SiMS that were immobilized on the glass surface were distributed with standard deviation of 16.3 nm and 16.0 nm in the *x-* and *y-*axes, respectively (Figure 2B). CF680R-actin SiMS incorporated into the lamellipodial actin network moving along the retrograde actin flow as observed with EGFP-actin and DL549-actin [15,16]. CF680R-actin enabled the measurement of the retrograde flow velocity of actin SiMS that had a short lifetime less than 10 s (Figure 2C). In the analysis of CF680R-actin, displacement over the distance of 200–300 nm for 3–5 s was sufficient for reliable measurement of the actin SiMS velocity.

**Figure 3:**
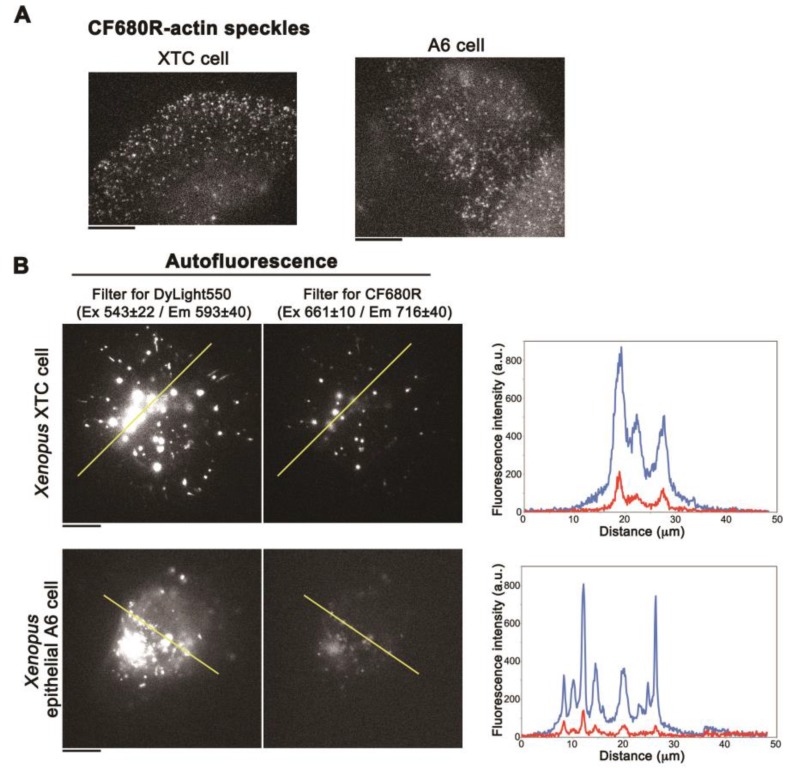
(**A**) Images of CF680R-actin SiMS in cell periphery of XTC cells (left) or A6 cells (right). The acquisition condition is with the custom filter set for CF680R-actin (an exciter filter: 661 ± 10 nm, an emitter filter: 716 ± 40 nm and a dichroic beamsplitter: 685 nm), the 75 W xenon illumination attenuated by a 12% neutral density filter, and a 1 s exposure time. Bar = 10 µm; (**B**) Autofluorescence images in an intact *Xenopus* XTC cell (upper) or A6 cell (lower) acquired under the conditions for DL550-actin SiMS (left) or CF680R-actin SiMS (right). The acquisition condition for DL550-actin SiMS is with the filter set for DL550-actin (Semrock TRITIC-B single-band filter set composed of an exciter filter: 543 ± 20 nm, an emitter filter: 593 ± 40 nm and a dichroic beamsplitter: 562 nm), the 75 W xenon illumination attenuated by a 12% neutral density filter and a 1 s exposure time. The acquisition condition for CF680R-actin SiMS is the same as in (**A**). The right graphs show fluorescence intensity measured by line-scan analysis along the yellow line in the images. Blue lines: Autofluorescence detected with the filter set for DL550. Red lines: Autofluorescence detected with the filter set for CF680R. Bar = 10 µm. The data shown are representative of four independent experiments.

CF680R-actin SiMS incorporated into the actin meshwork in lamellipodia (Figure 3A) as is the case with DL549-actin [16]. Next, we compared the fluorescence intensity ratio of autofluorescence and fluorescent actin in XTC cells and *Xenopus* A6 kidney epithelial cells. We acquired autofluorescence under the conditions identical to those used for CF680R-actin SiMS (see the legend of Figure 3A) and DL550-actin SiMS (see the legend of Figure 3B). The brightness of DL550-actin SiMS (Supplementary Figure S1) was similar to CF680R-actin SiMS (Figure 3A) under the acquisition conditions for each actin probes described in the legend of Figure 3A,B. In A6 cells, the fluorescent intensities of DL550-actin SiMS and CF680R-actin were 1155 ± 246.1 and 1001 ± 221.1 (mean ± SD, arbitrary unit), respectively. In either XTC cells or A6 cells, autofluorescence in the cell body was 5- to 6-fold lower with the filter set for CF680R-actin than with the filter set for DL550-actin (Figure 3B). In A6 cells, the intensity ratio of autofluorescence and SiMS brightness with CF680R-actin was reduced to approximately 13% of that with DL550-actin.

**Figure 4:**
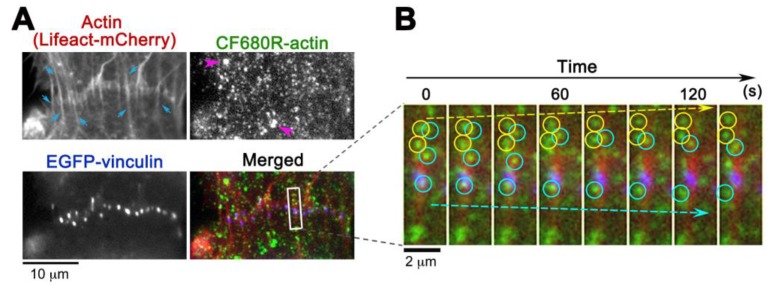
Observation of CF680R-actin SiMS in actin bundles at cell-to-cell boundaries of XTC cells. (**A**) CF680R-actin co-assembles to actin bundles (blue arrows) visualized by Lifeact-mCherry. The actin bundles are originated from punctate adherens junctions marked by EGFP-vinculin. Pink arrowheads in the CF680R-actin image indicate fluorescent organelle-like structures, which is apparently an artifact of electroporation; (**B**) Time-lapse images paneled at 20 s intervals in the area (rectangle) are shown on the right. Actin bundle-associated CF680R-actin SiMS moving upward (yellow arrow) and downward (blue arrow) are indicated by yellow (upward) or blue (downward) circles. Time-lapse images of CF680R-actin SiMS (green) are overlaid with the static images of Lifeact-mCherry (red) and EGFP-vinculin (blue).

CF680R-actin with green and red fluorescent probes extends application of eSiMS microscopy to three-color imaging. At the cell-to-cell boundaries of XTC cells, CF680R-actin SiMS incorporated into actin bundles that were visualized with Lifeact-mCherry (Figure 4A). These actin bundles were originated from punctate adherens junctions marked with EGFP-vinculin (Figure 4A). In time-lapse images, CF680R-actin SiMS associating with the actin bundles moved toward the upper cell (yellow circles and arrow) or the lower cell (blue circles and arrow) at the cell-to-cell boundaries (Figure 4B). Interestingly, actin speckles above the punctate adherens junction in the images moved in two opposite directions: both upward and downward (Figure 4B). At sites of cell–cell contact, actin filaments are considered to be pulled toward the cell center by actomyosin contraction [24]. Our observation suggests coexistence of overlapping actin structures pulled in two distinct directions. Such actin structure might be related to adhesion zippers derived from filopodia of opposing cells [25]. Another possibility is that the actin speckles observed might have experienced a flow-like movement of actin at the tilted cell–cell junctions [26].

CF680R-actin labeled bright organelle-like structures (Figure 4, pink arrows), as is the case with DL550-actin [16]. We speculate that the structure is an artifact arising from endocytosis of fluorescently labeled actin during electroporation [16,17]. Such organelle-like structures are markedly larger and brighter than SiMS and they move in stochastically changing directions [16]. We eliminated the structure from analysis.

**Figure 5:**
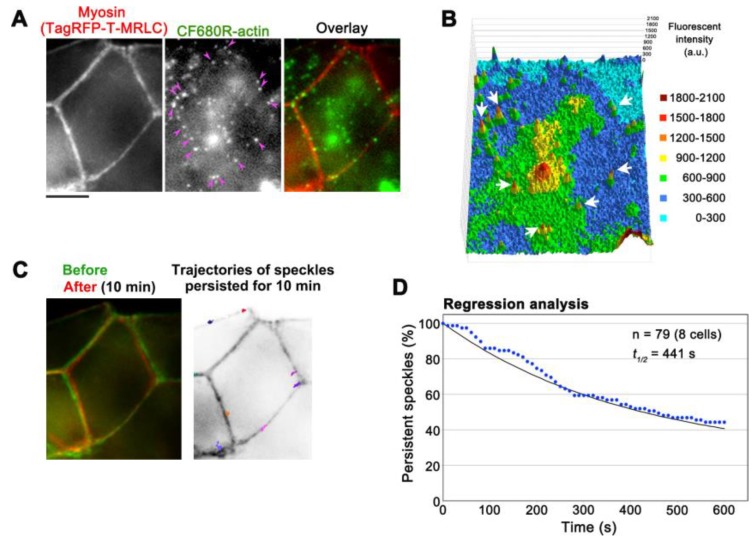
Observation of CF680R-actin SiMS in actomyosin bundles associated with adherens junctions (AJs) in A6 cells. (**A**) Representative images of actomyosin bundles visualized by TagRFP-T-myosin regulatory light chain (MRLC, left), CF680R-actin SiMS (middle), and the merged image (right). Pink arrowheads indicate CF680R-actin SiMS in actomyosin bundles associated with AJs. Bar = 5 µm; (**B**) The middle image of (**A**) is shown in a 3D profile, in which the *z*-axis represents the fluorescence intensity of CF680R-actin. The fluorescence intensity of each pixel is shown with colors as indicated. White arrows indicate CF680R-actin SiMS; (**C**) Left: A merged image of actomyosin bundles associated with AJs visualized by TagRFP-T-MRLC before (green) and after (red) a 10 min time-lapse imaging of CF680R-SiMS. Right: Trajectories of CF680R-actin SiMS in actomyosin bundles associated with AJs. The trajectories of actin SiMS that persisted from the first image to the last image of a 10 min time-lapse imaging were indicated with colored lines and overlaid with the inverted image TagRFP-T-MRLC (before); (**D**) Speckle regression analysis of CF680R-actin SiMS in actomyosin bundles associated with AJs in eight cells. The black line indicates that the one-phase exponential curve fitted to the data (blue dots) gives 441 s for the half-life of SiMS.

The low background of CF680R-actin provides an opportunity to monitor actin structures in deeper cellular areas. In combination with epi-fluorescent microscopes, either SiMS using EGFP-tagged proteins or eSiMS employing DL550-actin has been mainly used to observe thin cell peripheral areas to avoid autofluorescence background. Using CF680R-actin, we visualized actin SiMS in actomyosin bundles associating with adherens junctions (AJs) of A6 cells forming epithelial monolayers with epi-fluorescent microscopy (Figure 5A,B, Video S3). In A6 cell monolayers, AJs are located at 3.5–4 μm from basal surfaces [23] and can be monitored within a single focal plane with a 60× silicone oil objective manufactured by Olympus. The silicone oil objective is suitable to observe deep cellular structures by reducing spherical aberration caused by refractive index (RI) mismatch. The RI of silicone oil for immersion medium is close to that of living tissues, which improves optical performance for imaging in thick samples. At the electron microscopic level, actin filament bundles running parallel to the plasma membrane have been observed in the zonula AJs [27]. CF680R-actin was presumably incorporated into such actin bundles. In the actin bundles, CF680R-actin SiMS stayed in almost the same position in a 10 min time window (Figure 5C). The average displacement of CF680R-actin SiMS for 10 min was 0.61 ± 0.36 µm (n = 19 speckles). The slight movements of CF680R-actin SiMS in actin bundles appeared to accompany the changes in cell shape (Figure 5C).

We analyzed the disassembly rate of actin bundles by regression analysis of CF680R-actin SiMS, in which the surviving fraction of preexisting actin speckle is followed [15,22]. Our data revealed that actin filaments in actomyosin bundles associated with AJs disassembled with a half-life of 441 s (Figure 5D). As for actin structures in XTC cells, the average filament lifetime is 32.9 s in lamellipodia and 79.3 s in filopodia whereas the majority of actin filaments in actin stress fibers disassemble with a half-life of 5 min [16]. Therefore, the turnover of actin filaments in actomyosin bundles in AJs is relatively slow among actin structures observed in cultured cells.

One of the advantages of eSiMS for actin is that fluorescently labeled actin on lysine retains the ability to incorporate into actin filaments assembled by formin-homology proteins (formins) including mDia1 and mDia2 [16]. Formins are the major family of actin nucleators that also accelerate actin elongation [28,29,30], and play critical roles in the formation and stabilization of AJs formed in cultured cell systems and during Drosophila embryogenesis [31]. There are concerns about the usage of EGFP-actin for monitoring formin-based actin structures because several observations including our own indicated that EGFP-actin is not efficiently incorporated into actin filaments assembled by formins [16,32]. Therefore, the usage of EGFP-actin for the measurement of the actin turnover associated with AJs by fluorescence recovery after photobleaching (FRAP) in the previous studies [33,34] may have partially hindered monitoring of the formin-based actin turnover. In the present study, we were able to measure the turnover rate of actin filaments associated with AJs including formin-based actin networks for the first time.

## 4. Conclusions

In the present study, we introduced the new NIR emissive CF680R-actin probe for eSiMS microscopy. In live cells, single molecules of CF680R-actin can be visualized with low autofluorescence background, which is approximately 13% of eSiMS imaging with DL550-actin. In addition, CF680R-actin is considerably bright and photostable, which allows imaging of single molecules at high spatiotemporal resolution. These favorable properties of CF680R-actin would assist eSiMS imaging of deep cellular actin structures. Furthermore, CF680R-actin extends application of eSiMS microscopy to three-color single-molecule imaging. Its combination with green and red fluorescent probes will help researchers to visualize the molecular behavior of multiple cellular structures simultaneously in the same cell.

## Supplementary Materials

The following are available online at http://www.mdpi.com/1424-8220/17/7/1545/s1, Figure S1: Images of DL550-actin SiMS in cell periphery of XTC cell (left) or A6 cell (right) acquired under the acquisition condition for DL550-actin SiMS described in the legend of Figure 3B. Bar = 5 μm, Video S1: Intracellular photostability of fluorescent dye-labeled actin probes (Figure 1), Video S2: Photostability of CF680R-actin under attenuated xenon excitation (Figure 2A), Video S3: Time-lapse movie of SiMS of CF680R-actin in AJ of A6 cells (Figure 5A).
