# Supplementary material for: An Infrared Actin Probe for Deep-Cell Electroporation-Based Single-Molecule Speckle (eSiMS) Microscopy
Source: Sensors (Basel). 2017 Jul 1;17(7):1545. doi: 10.3390/s17071545 (PMC5539718; doi:10.3390/s17071545)
Supplement: Supplementary file 1 [file sensors-17-01545-s001.zip › sensors-196942 - supplymentary for pub/Supplementary Figure S1.docx]

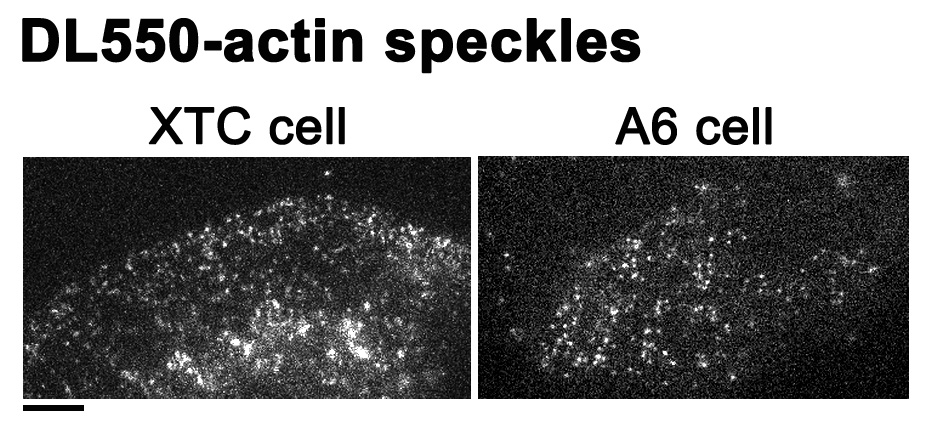


**Figure S1.** Images of DL550-actin SiMS in cell periphery of XTC cell (left) or A6 cell (right) acquired under the acquisition condition for DL550-actin SiMS described in the legend of Fig. 3B. Bar = 5 μm.
